# Antioxidant Activity of Three Honey Samples in relation with Their Biochemical Components

**DOI:** 10.1155/2013/313798

**Published:** 2013-08-21

**Authors:** Lee Suan Chua, Norul Liza A. Rahaman, Nur Ardawati Adnan, Ti Tjih Eddie Tan

**Affiliations:** ^1^Metabolites Profiling Laboratory, Institute of Bioproduct Development, Universiti Teknologi Malaysia, UTM Skudai, 81310 Johor Bahru, Malaysia; ^2^Food Technology Programme, Universiti Teknologi MARA, Kuala Pilah Campus, 72000 Kuala Pilah, Negeri Sembilan, Malaysia

## Abstract

The antioxidant activities based on the free radical scavenging, reducing power, and bleaching inhibition were investigated for the three commonly used honeys in Malaysia, namely, tualang, gelam, and acacia honey. The antioxidant capacity of the honey samples was correlated with their biochemical constituents such as total phenol, total flavonoid content, and total water-soluble vitamins (vitamin B1, B2, B3, B9, B12, and vitamin C). The total flavonoid content of honey samples was strongly correlated with the three antioxidative processes (*r* = 0.9276–0.9910). In contrast, the total water-soluble vitamins was found to be well correlated with the free radical scavenging activity (*r* = 0.8226). Vitamin B3 was likely to be in the highest concentration, which covered for 69–80% of the total vitamin content. A number of five phenolic acids, three flavonoids, and two organic acids had also been detected from the honey samples using UPLC-MS/MS, without sugar-removal procedure.

## 1. Introduction

Honey is well known as a natural dietary antioxidant. The components responsible for the redox properties of honey are likely to be phenolic acids, flavonoids, vitamins, and enzymes, as well as a small amount of mineral content, particularly copper and iron [1, 2]. However, little is known about the antioxidant capacity and the mechanism involved by each biochemical component either through reducing power or radical scavenging activity of honey from tropical countries. It might also be attributed to the combined activity of these minor components through synergistic effects [3, 4].

Numerous studies have reported that most chronic diseases such as cancer, coronary, and neurological degeneration are a consequence of oxidative damage. It is also proven that the therapeutic potential of honey is always associated with antioxidant capacity against reactive oxygen species [5]. Therefore, in recent years, studies have been focused on the composition of honeys and their biological properties such as antioxidant [6], anti-inflammatory [7], and antimicrobial activities [8] in wound healing [9], as well as in the treatment of skin ulcers [10] and gastrointestinal disorders [11]. 

To our knowledge, there is no official method available for the determination of antioxidant activity in honey samples [12]. The commonly used antioxidant assays include DPPH (free radical scavenging activity), FRAP (ferric reducing/antioxidant power), *β*-carotene bleaching assay, ORAC (oxygen radical absorbance capacity), ascorbic acid antioxidant content (AEAC), and Trolox equivalent antioxidant activity (TEAC). Each assay has its advantages and disadvantages. DPPH free radicals are reported to be unaffected by certain side reactions such as metal ion chelation and enzyme inhibition [13]. Furthermore, honey contains abundant free radical scavengers, which are able to reduce the imbalance between free radical production and antioxidant level [14]. The high amount of reducing sugars (>65%) such as glucose and fructose in honey could contribute to higher reducing antioxidant power in the FRAP method, which would lead to positive error in the determination of antioxidant activity [5]. 

Polyphenols are currently of particular interest to medical and food nutrition research, mainly because of their functional properties. Besides being radical scavenger, polyphenol could be an effective immune modulator and hormone action inhibitor [15]. Polyphenols are believed to be potent scavengers of peroxyl radicals, mainly because of the presence of high mobility of hydrogens in their molecular structures [6]. Of the polyphenols, phenolic acids are likely to be the major group in honey. They have also been reported to affect the flavor [16] and physical appearance of honey, particularly honey colour [17, 18]. Interestingly, they have been given considerable attention to be an eligible parameter for honey quality assessment [6], as well as for honey marker identification, with the help of the advancement of liquid chromatography and mass spectrometry technology nowadays.

Because of the great variation in honey composition, the biological activities exhibited by the honey samples varied according to the geographical and botanical origin of honey, while processing and storage condition might affect the biological activities only to a minor degree [3]. Therefore, this study has investigated the comparative antioxidant property of the three commonly used honeys in Malaysia in relation with their biochemical components. Since honey is an aqoueus-based foodstuff, only water-soluble vitamins were determined in this study.

## 2. Experimental

### 2.1. Chemicals and Reagents

Formic acid, acetic acid, phosphoric acid, sodium carbonate, iron (III) chloride, USA and aluminium chloride were obtained from Fisher Scientific (Pittsburgh, PA, USA). Coomassie Brilliant Blue G-250 (CBB), butylated hydroxytoluene, and HPLC-grade of methanol (MeOH), hexane, acetone, chloroform and acetonitrile (CH_3_CN) were obtained from Merck (Darmstadt, Germany). 18.2 MΩ·cm water was produced from a Barnstead NANOpure Diamond water purification system (Thermo, Waltham, MA, USA). Folin-Ciocalteau reagent, 1,1-diphenyl-2-picrylhydrazyl (DPPH), vitamin B1 (≥99%), vitamin B2 (≥98%), vitamin B3 (≥99.5%), vitamin B9 (≥97%), vitamin B12 (≥98.5%), vitamin C (≥99%), *β*-carotene (≥93%), linoleic acid (≥99%), Trolox (97%), Tween 20, and 2,4,6-tripyridyl-s-triazine (≥98%) were sourced from Sigma-Aldrich (St. Louis, MO, USA). The standard gallic acid (98%) and rutin (97%) were obtained from Acros Organics (Pittsburgh, PA, USA). Bradford reagent and bovine serum albumin (BSA) standard were obtained from Bio-Rad Laboratories, Hercules, USA. All reagents were of analytical grade unless otherwise stated.

### 2.2. Honey Samples

The three most commonly used honeys in Malaysia, tualang, gelam, and acacia honey, were harvested by local suppliers between April and June 2011. The results were expressed as mean of the data collected in triplicate from different months for each honey. Tualang and gelam honeys were obtained from Federal Agriculture Marketing Authority (FAMA), Kedah, whereas acacia honey was supplied by MB An-Nur Apiary, Johor. 

### 2.3. Total Phenolic Content

The total phenolic content (TPC) of honey samples was analysed by using Folin-Ciocalteu reagent, based on the method described by Singleton et al. (1998) [19] with some modification. Honey solution (0.5 mL) was mixed with 2.5 mL of Folin-Ciocalteau reagent (2N) and incubated for 5 min. Subsequently, 2 mL of sodium carbonate solution (75 g/L) was added into the honey solution and incubated for another 2 h at 25°C. After incubation, the absorbance of the solution was measured at 765 nm by using a UV-Visible spectrophotometer (Perkin-Elmer Lambda 25, Waltham, MA, USA). Gallic acid (0–1000 mg/L) was used as a standard chemical for calibration curve preparation. The TPC was reported as mean value of triplicate assays and expressed as milligram of gallic acid equivalent (GAE) in gram of honey.

### 2.4. Total Flavonoid Content

The total flavonoid content (TFC) of honey samples was determined based on the method of Isla et al. (2011) [18], with some modification. A 5 mL of honey solution (0.1 g/mL) was mixed with 5 mL of 2% aluminium chloride (AlCl_3_). A flavonoid-aluminium complex was formed after 10 min of incubation time at 25°C. The formation of the complex was measured at 415 nm by using an UV-Visible spectrophotometer. Rutin (0–100 mg/L) was used as a standard chemical for calibration curve preparation. The TFC was reported as mean value of triplicate assays and expressed as milligram of rutin equivalent (RE) in gram of honey.

### 2.5. Free Radical Scavenging Activity

The free radical scavenging activity of honey samples was determined by the 1,1-diphenyl-2-picrylhydrazyl (DPPH) assay as described by Isla et al. (2011) [18], with minor modification. The DPPH^∙^ solution (20 mg/L) was prepared by dissolving 2 mg of DPPH^∙^ in methanol (100 mL). A 0.75 mL of methanolic honey solution at different concentrations, ranging from 20 to 40 mg/mL, was added to 1.5 mL of DPPH^∙^ solution. The absorbance was measured at 517 nm after 15 min of incubation at 25°C. Ascorbic acid was used as positive control. The ability to scavenge the DPPH^∙^ was calculated using (1), where *A*_control_ and *A*_sample_ are the absorbances of control and sample, respectively. The concentration of honey sample required to scavenge 50% of DPPH^∙^ (EC_50_) was determined based on the ascorbic acid calibration curve (0–10 mg/L) [20]. The experiment was performed in triplicate:

(1) $$\begin{array}{c} \text{DPPH}\;\text{scavenging}\;\text{activity}\;\left(\%\right)=\frac{A_{\text{control}}-A_{\text{sample}}}{A_{\text{control}}}\times 100. \end{array}$$

### 2.6. Ferric Reducing/Antioxidant Power Assay

The reducing power of honey samples was determined based on the method described by Benzie and Strain (1996) [21] with minor modification. The principle of this method is based on the reduction of a ferric 2,4,6-tripyridyl-s-triazine complex (Fe^3+^-TPTZ) to its ferrous, coloured form (Fe^2+^-TPTZ) in the presence of antioxidants. The FRAP reagent was prepared by mixing 2.5 mL of a 10 mM TPTZ (2,4,6-tripyridyl-s-triazine) solution in 40 mM HCl, 2.5 mL of 20 mM FeCl_3_, and 25 mL of 0.3 M acetate buffer at the pH of 3.6. It was prepared daily and was warmed to 37°C before use. Honey (1 g) was well dissolved in 10 mL of n-hexane-acetone mixture (6 : 4), and the honey solution was filtered through Whatman number 4 filter paper. An aliquot of 200 *μ*L of honey solution was mixed with 1.8 mL of FRAP reagent, and the absorbance of the reagent mixture was measured spectrophotometrically at 593 nm after incubation for 10 min. Trolox was used for the calibration curve, and the results were expressed as milligram of Trolox equivalent (TE) per 100 gram of honey. 

### 2.7. *β*-Carotene Bleaching Assay

The antioxidant activity of honey was evaluated based on the *β*-carotene linoleate model system as described by Ferreira et al. (2009) [5] with modification. 2 mL of *β*-carotene (0.2 g/L) in chloroform, 0.02 mL of linoleic acid, and 0.2 mL of Tween 20 were transferred into a 100 mL round bottom flask. A 0.2 mL honey solution was added into the mixture. After evaporation to dryness under vacuum at room temperature, 50 mL of distilled water was added into the flask. The mixture was agitated vigorously to form an emulsion. A 2 mL aliquot of the emulsion was transferred to another test tube and immediately placed in water bath at 50°C. The absorbance of the sample was measured every 20 minutes for 2 hours at 470 nm using a UV-Visible spectrophotometer. Butylated hydroxytoluene (200 mg/mL) was used as standard chemical for the construction of calibration curve. The results were expressed as mean of triplicate assay. The *β*-carotene bleaching activity (CBI) was calculated as inhibition relative to control using (2) where *B*_control_ and *B*_sample_ were the bleaching rates of *β*-carotene in control and sample, respectively [22]:

(2) $$\begin{array}{c} \text{CBI}\;\left(\%\right)=\frac{B_{\text{control}}-B_{\text{sample}}}{B_{\text{control}}}\times 100. \end{array}$$

### 2.8. Water-Soluble Vitamins Analysis

Water-soluble vitamins in honey samples were determined by using the method reported by Ciulu et al. (2011) [23], with some minor modification. A total of six water-soluble vitamins such as vitamin B1 (thiamin), B2 (riboflavin), B3 (niacinamide), B9 (folic acid), B12 (cobalamin), and vitamin C (ascorbic acid) were quantified using a reversed phase HPLC (Waters 600, MA, USA) connected with a PDA detector (Waters 996, MA, USA). The stock solution of the vitamin standard mixture was prepared by dissolving 2.5 mg of vitamin B1, B2, B9, B12 and 20 mg of vitamin C into 50 mL of phosphate buffer (1 M, pH 5.5) and 4 mL of sodium hydroxide (2 mol/L) in a 100 mL volumetric flask. Only the stock solution of standard vitamin B3 (5 mg) was individually prepared. The stock solutions were then topped up to the mark with ultrapure water and kept at 4°C. A serial concentration of the standard solutions (0–100 mg/L) was prepared freshly for calibration curve construction. 

A 2 g of homogenised honey sample was weighted and dissolved in 2 mL of ultrapure water. Then, 0.2 mL of sodium hydroxide and 2.5 mL of phosphate buffer were added and topped up to 5 mL. The honey solution was filtered with nylon filter (0.45 *μ*m, 13 mm) before injection. A Synergy C18 column (5 *μ*m, 250 × 4.6 mm) from Phenomenex (CA, USA) was used for the separation with the mobile phases of trifluoroacetic acid (0.025% v/v) aqueous solution as solution A and acetonitrile (100% v/v) as solution B at a constant flow rate of 1.0 mL/min. The gradient of the mobile phases was as follow: 100–75% A in 11 min at 254 nm; 75–55% A in 8 min at 210 nm; 55–100% A in 3 min at 210 nm, and then equilibrate the column in 100% A for 5 min. The total run time was 27 min with the flow rate 1 mL/min. Sample injection volume was 10 *μ*L. 

### 2.9. Polyphenols Determination by UPLC-MS/MS

An ultraperformance liquid chromatography (UPLC), Waters Acquity (Milford, MA, USA), system was coupled with a triple-quadrupole-linear ion trap tandem mass spectrometer (Applied Biosystems 4000 Q TRAP; Life Technologies Corporation, Carlsbad, CA, USA) with an electrospray ionisation (ESI) source. A C18 reversed phase Acquity column (1.7 *μ*m, 150 mm × 4.6 mm) protected by a guard column was used throughout this study.

The mobile phase was a binary solvent system consisting of solvent A (water with 0.1% formic acid) and solvent B (CH_3_CN). The UPLC gradient for mass screening was 0–5 min, 90% A; 5–15 min, 90–10% A; 15–20 min, 10% A; 20–25 min, 10–90% A; 25–30 min, 90% A for final washing and equilibration of the column for the next run. The UPLC gradient for the detection of target mass was similar to mass screening profile, except that the total run time was shortened to 15 min. The flow rate was 0.25 mL/min, and the injection volume was 5 *μ*L. All samples were filtered with 0.2 *μ*m nylon membrane filter prior to injection.

The mass spectra were acquired from *m*/*z* 100–1000 with a 20 ms ion accumulation time. All mass spectrometric data were acquired in positive ionisation mode. The capillary and voltage of the ESI source were maintained at 400°C and −4.5 kV, respectively. All other parameters were as follows: nitrogen was used as ion source gas for nebulisation, 40 psi; for drying solvent, 40 psi; curtain gas, 10 psi; collision gas, high; declustering potential, −40 V; and collision exit energy, −10 V. The scan rate was 1000 amu/s. Data acquisition and data processing were performed using Analyst 1.4.2. MS Fragmenter 12.0 (Advanced Chemistry Development, Toronto, Canada) was used to predict compound fragmentation.

## 3. Results and Discussion

### 3.1. TPC and TFC

TPC is considered as a fast and simple method to measure the total phenol in complex matrix like honey. Al et al. (2009) [24] reported that this method was sensitive enough for total phenol estimation in honey samples. In the present study, the TPC of honey samples was in the narrow range from 110.394 to 196.500 mg GAE/100 g honey (Table 1). The TPC results were higher than commercial Indian honeys (47–98 mg GAE/100 g honey) [25] and Argentinean northwest honeys (18.730–107.213 mg GAE/100 g honey) [18], as well as Burkina Fasan honeys (32.59–114.75 mg GAE/100 g honey) [2]. 

Total phenol was also compared to the TPC value reported for the similar type of honey from different countries. It was found that acacia honey from Malaysia showed higher TPC value (196.500 mg GAE/100 g honey) than acacia honeys from Germany (4.6 mg/100 g honey) [3], from Romania (2.00–39.00 mg GAE/100 g honey) [24], from Burkina Faso (93.43 mg GAE/100 g honey) [2], and from Slovenia (4.48 mg GAE/100 g honey) [12]. 

To the best of our knowledge, there are two widely used spectrophotometric methods to determine total flavonoid in honey samples. Both methods measure the formation of coloured complex substances quantitatively, after reacting flavonoids with aluminium ion (III) or 2,4-dinitrophenylhydrazine (DNP). Since DNP method has been reported as less reliable [26], the TFC of honey samples was determined based on the method of aluminium chloride (AlCl_3_), which was specific for flavones and flavonols [17]. Furthermore, Iurlina et al. (2009) [27] reported that the predominant flavonoid in honey samples was from the group of flavonols (45% quercetin). 

The TFC assay found that the honey samples in this study exhibited higher TFC values (18.511–32.866 mgRE/100 g honey) than those values reported for Portuguese heater honey (0.06–0.50 mg/100 g honey) and Spanish rosemary honey (0.50–2.00 mg/100 g honey) [28]. TFC of this study was approximately covered for 15–20% of the total phenols (Table 1).

### 3.2. Water-Soluble Vitamins in Honey

In addition to polyphenols, the antioxidant activity of honey might be also attributed to the presence of several water-soluble vitamins such as vitamin B1 (thiamine), B2 (riboflavin), B3 (nicotinic acid), B9 (folic acid), B12 (cyanocobalamin), and vitamin C as presented in Table 2. The RP-HPLC method proved the presence of these vitamins in the honey samples with vitamin B3 as the highest content, approximately covered for 62–80% of the total water-soluble vitamin content. The validation data, namely, sensitivity in terms of limit of detection (LOD) and limit of quantification (LOQ), and linearity within the range of 0–250 mg/kg with correlation coefficients (*r* ^2^) more than 0.998 were determined (Table 2). Significantly, the vitamin content (B2, B3, B9, and C) in acacia honey was higher than the results reported for Italian acacia honey by Ciulu et al. (2011) [23]. The vitamin C content in all honey samples was also higher than the usual level of vitamin C concentration, 50 mg/kg honey [29]. Somehow, vitamins in honey are sensitive to processing and storage. Gheldof et al. (2002) [3] reported that the loss of vitamin C in the selected commercial honey most likely was due to the processing and storage. 

### 3.3. Polyphenols and Antioxidant Activity of Honey

**Figure 1:**
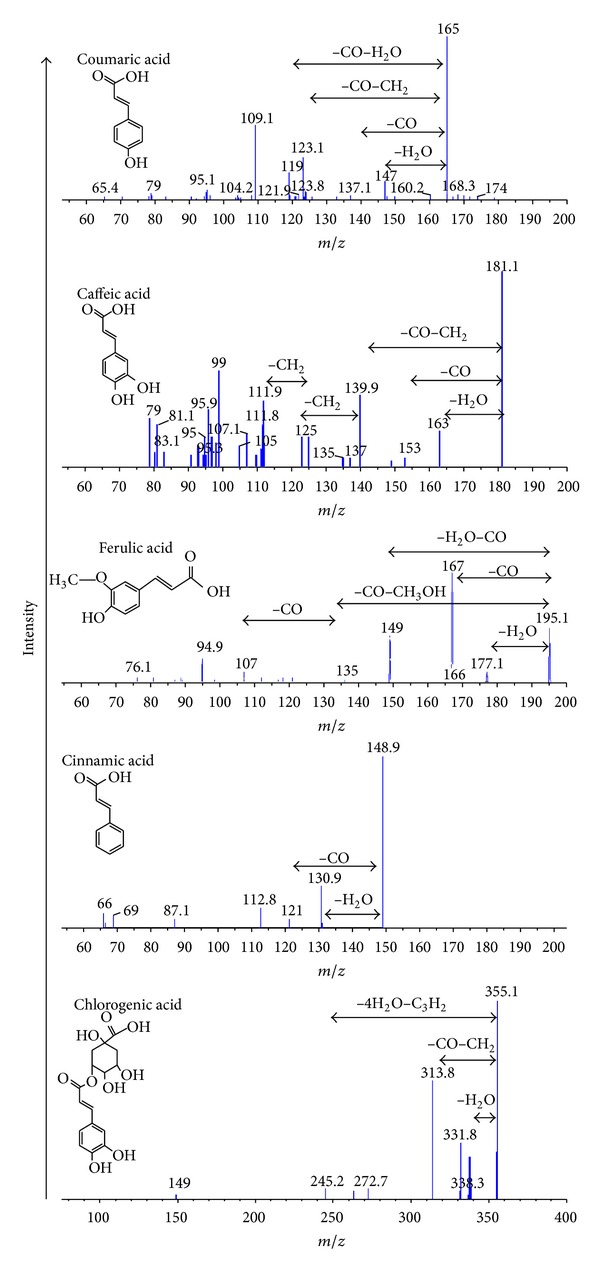
Mass spectra of phenolic acids at the positive mode of enhanced product ion scan.

A number of five phenolic acids had been detected from the honey samples by using multiple reaction monitoring (MRM) and enhanced product ion (EPI) scan modes of UPLC-MS/MS method (Table 3). The phenolic acids were detected at the positive mode of mass screening and then confirmed by enhanced product ion scan. Besides phenolic acids, the other detected flavonoids include flavone (quercetin) and flavanone (pinobanksin-3-O-butyrate and pinobanksin-3-O-propionate). It is important to highlight that pinobanksin has been demonstrated to be a potent antioxidant [30]. This hybrid method offers obvious advantages, in terms of sensitivity and selectivity, without the need for standard chemical, sugar separation, and clean-up procedures. The compounds were identified based on the characteristic ions as structural information from the fragmentation pattern. The positive mode of mass spectra for the detected phenolic acids is presented in Figure 1.

Another two commonly found organic acids in honey such as fumaric and gluconic acids were also detected in this study. Even though organic acids are just covered for less than 0.5% of honey composition, the presence of organic acids might affect the honey flavor and stability against microorganisms. These organic acids might also contribute to the antioxidant capacity of honey by chelating with metals, thus synergistically enhancing the antioxidative action of phenolics [31]. According to Cherchi et al. (1994) [32], gluconic acid is the predominant organic acid in honey which could achieve up to 50-fold higher concentration than other acids. 

The scavenging activity of honey samples had been measured by using DPPH assay, and ascorbic acid was used as positive control. The unpaired electron of DPPH forms a pair with a hydrogen donated by free radical scavenging antioxidant from honey and thus converting the purple coloured odd electron DPPH to its reduced form in yellow. The degree of decolourization would be measured by UV-Visible spectrophotometer in order to determine the scavenging activity of honey. It was found that the EC_50_ of ascorbic acid was about a few thousands lower than all honey samples. The lower the EC_50_ value the higher the scavenging capacity of honey, because it requires lesser amount of radical scavenger from the honey to reduce DPPH.  Table 4 indicates that gelam honey has the highest scavenging activity, followed by acacia and tualang honey samples.

The findings were in contradiction with the previous study conducted by Kishore et al. (2011) [14]. They reported that tualang honey (EC_50_ = 5.8 mg/mL) had the highest scavenging activity compared to other honey samples, including gelam honey (EC_50_ = 6.68 mg/mL). However, the scavenging activity of gelam honey was found to be higher than tualang honey in this study. Hence, gelam honey was about three times more efficient than tualang honey as free radical scavenger. The contradiction was also observed in the results of TPC and TFC, even though the honey samples were collected from the similar supplier, FAMA, Kedah in the study of Kishore et al. (2011) [14]. Most probably, the variance was contributed by the difference in honey harvesting time. 

In term of reducing power, tualang honey has also expressed the lowest content of reductant against oxidative damage compared to gelam and acacia honey (Table 4). The antioxidant capacity of tualang honey based on the inhibition of *β*-carotene bleaching activity had also the lowest value (35.81%) as exhibited in the assay of DPPH and FRAP. This bleaching assay describes the ability of antioxidants in honey to reduce the chromophore degradation of *β*-carotene throughout the duration of the assay [20]. However, the bleaching inhibition of acacia honey (74.66%) was higher than gelam honey (67.41%). It means that gelam honey showed higher antioxidant activity in term of radical scavenging and ferric-reducing mechanism than acacia honey but lower in term of *β*-carotene bleaching activity. 

### 3.4. Correlation between Antioxidant Activity and Its Biochemical Component

Statistical tool can be considered as a useful complimentary approach to investigate the relationship between the antioxidant activities of honey and its biochemical composition. From the table of correlation matrix (Table 5), TPC was strongly correlated with FRAP and *β*-carotene bleaching activity, but not for DPPH assay. However, TFC was more likely well correlated with all the three antioxidant assays with different mechanisms. In contrast, the total water-soluble vitamin content of honey samples in this study was only correlated with the scavenging activity. The water-soluble vitamins in honey might act as radical scavenger to minimize oxidation process. The scavenging activity of the water-soluble vitamins was likely to be higher than phenolic compounds but lower than flavonoids in the honey samples. 

## 4. Conclusions

 The total flavonoid content of honey samples was strongly correlated with the antioxidant activities such as DPPH free radical scavenging activity (*r* = 0.9276), ferric reducing antioxidant power (*r* = 0.9910), and *β*-carotene bleaching inhibition (*r* = 0.9508). However, the total phenol was only strongly correlated with ferric-reducing antioxidant power (*r* = 0.9033) and *β*-carotene bleaching inhibition (*r* = 0.9656). In contradiction with the correlation of total phenol, the total water-soluble vitamins was found to be well correlated with the free radical scavenging activity (*r* = 0.8226). From the fast screening method using UPLC-MS/MS, a few phenolic acids were detected from honey samples, in addition to flavone (quercetin) and flavanone (pinobanksin-3-O-butyrate and pinobanksin-3-O-propionate), as well as organic acids. Besides vitamins, these compounds were most probably the key constituents contributing to the antioxidant capacity of honey samples.

## Figures and Tables

**Table 1:** Total phenols and total flavonoids in honey samples.

Honey	^ a^TPC (mg GAE /100 g honey)		^ b^TFC (mg RE /100 g honey)		TFC/TPC
	Mean	^ c^SD	Mean	^ c^SD	
Tualang	110.394	3.829	18.511	2.803	0.168
Gelam	159.743	9.004	32.886	0.780	0.206
Acacia	196.500	6.323	30.741	2.886	0.156

^a^Total phenolic content in milligram of gallic acid equivalent per 100 gram of honey.

^b^Total flavonoid content in milligram of rutin equivalent per 100 gram of honey.

^c^Standard deviation.

**Table 2:** Water-soluble vitamins in honey samples.

Water-soluble vitamins	^ a^Rt	Sensitivity (mg/kg)		Linearity (mg/kg)		Tualang	Gelam	Acacia
	(min)	^ b^LOD	^ c^LOQ	Concentration	^ d^ *R* ^2^	Mean ± ^e^SD (mg/kg honey)		
Thiamine (vitamin B1)	3.10	3.51	10.62	0–75	0.999	<LOQ	13.85 ± 0.24	<LOQ
Riboflavin (vitamin B2)	14.69	1.80	5.44	0–75	0.999	<LOD	<LOQ	11.85 ± 3.90
Nicotinic acid (vitamin B3)	1.83	4.76	14.42	0–250	0.999	170.38 ± 9.60	355.38 ± 56.11	134.67 ± 3.53
Folic acid (vitamin B9)	13.74	4.35	13.17	0–75	0.998	<LOQ	<LOD	<LOD
Cyanocobalamin (vitamin B12)	14.03	1.02	3.10	0–75	0.999	<LOD	<LOQ	<LOD
Ascorbic acid (vitamin C)	3.95	10.13	30.71	0–200	1.000	52.20 ± 3.29	67.36 ± 10.48	62.80 ± 8.60

^a^Retention time in minute.

^b^Limit of detection (3.3 standard deviation/slope of the calibration curve).

^c^Limit of quantitation (10 standard deviation/slope of the calibration curve).

^d^Correlation coefficient.

^e^Standard deviation.

**Table 3:** Phenolic acids, flavonoids, and organic acids detected from honey samples.

Compound	^ a^Rt (min)	[M + H]+	^ b^Honey	Fragment ions
Phenolic acids				
Coumaric acid	1.39	165	T, G, A	165/147(–H_2_O)/137(–CO)/123(–CO–CH_2_)/119(–H_2_O–CO)/109/95/91/78
Caffeic acid	1.40	181	T, G, A	181/163(–H_2_O)/153(–CO)/139(–CO–CH_2_)/125(–CO–2CH_2_)/111(–CO–3CH_2_)/99/79
Ferulic acid	1.50	195	T, G, A	195/177(–H_2_O)/167(–CO)/149(–H_2_O–CO)/135(–CO–CH_3_OH)/107(–2CO–CH_3_OH)/95
Cinnamic acid	1.51	149	T, G, A	149/131(–H_2_O)/121(–CO)/112/87
Chlorogenic acid	8.09	355	T, G, A	355/338(–OH)/337(–H_2_O)/313(–CO–CH_2_)/272/245(–4H_2_O–C_3_H_2_)/149
Flavonoids				
Pinobanksin-3-*O-*propionate	7.20	329	T, G	329/311(–H_2_O)/293(–2H_2_O)/273/265(–CO)/245/179/167
Pinobanksin-3-*O-*butyrate	7.47	343	T	343/325(–H_2_O)/301/273/240
Quercetin	8.98	303	T, G, A	303/285(–H_2_O)/261(–C_2_H_2_O)/220/180(–^1,2^A)/152(–^1,3^A)/114/96
Organic acids				
Fumaric acid	0.90	117	T, G, A	117/116(–H)/99(–H_2_O)/89(–CO)/85/75/73(–CO_2_)
Gluconic acid	1.10	197	T, G, A	197/179(–H_2_O)/161(–2H_2_O)/151(–H_2_O–CO)/136(–H_2_O–CO–CH_3_)/119(–H_2_O–CO–CH_3_–OH)/105/95

^a^Retention time in minute.

^b^T: tualang; G: gelam; A: acacia.

**Table 4:** Antioxidant activity of honey samples.

Honey	^a^DPPH (mg AAE/100 g honey)		^ b^EC_50_	^c^FRAP (mg TE/100 g honey)		^ d^CBI (%)	Kinetic equation of CBI	*r* ^2^ (Kinetic equation)
	Mean	^ e^SD	(mg/mL)	Mean	^ e^SD			
Tualang	9.650	0.570	48.896	52.386	5.192	35.81	*y* = −8.94 × 10^−5^ *x* + 1.31 × 10^−2^	0.868
Gelam	50.169	5.541	15.681	82.529	5.032	67.41	*y* = −4.54 × 10^−5^ *x* + 1.23 × 10^−2^	0.858
Acacia	29.983	6.064	29.846	82.386	5.930	74.66	*y* = −3.53 × 10^−5^ *x* + 8.20 × 10^−3^	0.812

^a^1,1-diphenyl-2-picrylhydrazyl scavenging activity in milligram of ascorbic acid equivalent in a 100 g of honey.

^b^50% scavenging activity of ascorbic acid was 8.611 *µ*g/mL.

^c^Ferric reducing/antioxidant power in milligram of Trolox equivalent in a 100 g of honey.

^d^ *β*-carotene bleaching inhibition of butylated hydroxytoluene was 81.54%.

^e^Standard deviation of triplicate assay.

**Table 5:** Correlation matrix between antioxidant activity and biochemical component.

	^ a^TPC	^ b^TFC	^ c^TWSV	^ d^DPPH	^ e^FRAP	^ f^CBI
^ a^TPC	1					
^ b^TFC	0.8375*	1				
^ c^TWSV	0.0052*	0.5508	1			
^ d^DPPH	0.5728*	0.9276	0.8226	1		
^ e^FRAP	0.9033	0.9910*	0.4338*	0.8691	1	
^ f^CBI	0.9656*	0.9508	0.2649	0.7662	0.9838	1

*Significant difference at *P* < 0.05 (two-tail *t*-test).

^a^Total phenolic content.

^b^Total flavonoid content.

^c^Total water-soluble vitamins.

^d^1,1-diphenyl-2-picrylhydrazyl scavenging activity.

^e^Ferric-reducing antioxidant power.

^f^ *β*-carotene bleaching inhibition.
